# Effective control of tumor growth through spatial and temporal control of theranostic sodium iodide symporter (*NIS*) gene expression using a heat-inducible gene promoter in engineered mesenchymal stem cells

**DOI:** 10.7150/thno.41489

**Published:** 2020-03-15

**Authors:** Mariella Tutter, Christina Schug, Kathrin A. Schmohl, Sarah Urnauer, Nathalie Schwenk, Matteo Petrini, Wouter J. M. Lokerse, Christian Zach, Sibylle Ziegler, Peter Bartenstein, Wolfgang A. Weber, Ernst Wagner, Lars H. Lindner, Peter J. Nelson, Christine Spitzweg

**Affiliations:** 1 Department of Internal Medicine IV, University Hospital of Munich, LMU Munich, Munich, Germany; 2 Department of Internal Medicine III, University Hospital of Munich, LMU Munich, Munich, Germany; 3 Department of Nuclear Medicine, University Hospital of Munich, LMU Munich, Munich, Germany; 4 Department of Nuclear Medicine, Klinikum rechts der Isar der Technischen Universität München, Munich, Germany; 5 Department of Pharmacy, Center of Drug Research, Pharmaceutical Biotechnology, LMU Munich, Munich, Germany

**Keywords:** sodium iodide symporter, regional hyperthermia, mesenchymal stem cells, gene therapy, theranostics

## Abstract

**Purpose**: The tumor homing characteristics of mesenchymal stem cells (MSCs) make them attractive vehicles for the tumor-specific delivery of therapeutic agents, such as the sodium iodide symporter (NIS). NIS is a theranostic protein that allows non-invasive monitoring of the *in vivo* biodistribution of functional NIS expression by radioiodine imaging as well as the therapeutic application of ^131^I. To gain local and temporal control of transgene expression, and thereby improve tumor selectivity, we engineered MSCs to express the *NIS* gene under control of a heat-inducible HSP70B promoter (HSP70B-NIS-MSCs).

**Experimental Design**: NIS induction in heat-treated HSP70B-NIS-MSCs was verified by ^125^I uptake assay, RT-PCR, Western blot and immunofluorescence staining. HSP70B-NIS-MSCs were then injected i.v. into mice carrying subcutaneous hepatocellular carcinoma HuH7 xenografts, and hyperthermia (1 h at 41°C) was locally applied to the tumor. 0 - 72 h later radioiodine uptake was assessed by ^123^I-scintigraphy. The most effective uptake regime was then selected for ^131^I therapy.

**Results**: The HSP70B promoter showed low basal activity *in vitro* and was significantly induced in response to heat. *In vivo*, the highest tumoral iodine accumulation was seen 12 h after application of hyperthermia. HSP70B-NIS-MSC-mediated ^131^I therapy combined with hyperthermia resulted in a significantly reduced tumor growth with prolonged survival as compared to control groups.

**Conclusions**: The heat-inducible HSP70B promoter allows hyperthermia-induced spatial and temporal control of MSC-mediated theranostic *NIS* gene radiotherapy with efficient tumor-selective and temperature-dependent accumulation of radioiodine in heat-treated tumors.

## Introduction

The sodium iodide symporter (NIS) is a transmembrane glycoprotein that actively co-transports two sodium and one iodide ion across the plasma membrane into the cytoplasm of thyroid follicular cells (reviewed in 1). The ability to accumulate and store iodide is a characteristic of thyroid tissue and a prerequisite for thyroid hormone synthesis. This feature allows the efficient treatment/curing of thyroid cancer through the systemic administration of radioiodide 2. NIS-expressing target cells absorb β-emitting radioisotopes, such as ^131^I or ^188^Re, and drive cell death via β-particulate radiation of the expressing cell and the neighboring tissues through bystander effects as the decaying particles have a path length of up to 2.4 mm in tissue.

Genetically targeting NIS to non-thyroidal tumor tissues has opened the prospect of transferring standard clinical protocols for radioiodine imaging and therapy to a wide range of extra-thyroidal tumor entities 3. Following cloning of the *NIS* gene in 1996 4, initial experiments of *ex vivo NIS* gene transfer 5 and local *NIS* gene delivery by intratumoral injections have been described 6. Subsequently, a series of diverse approaches have been evaluated for the systemic *in vivo* gene transfer into non-thyroidal tumors using viruses, nanoparticles or mesenchymal stem cells (MSCs) as carriers 7-28. To this end, adoptively applied MSCs have been demonstrated to exhibit an innate tumor tropism and have been extensively studied as potential tumor-selective gene transfer vehicles including progressing to clinical studies 29-44. Our group initially demonstrated the efficient transfer of functional *NIS* expression with accompanying therapeutic effects using MSCs transfected with *NIS* under the control of the constitutively active CMV-promoter 34. As a next step, to reduce potential non-tumor side effects by enhancing tumor-selective NIS expression, we studied the potential use of the tumor stroma-induced CCL5 (RANTES) gene promoter, which allowed a robust tumoral iodine accumulation in experimental tumors in mice leading to significantly reduced tumor growth and prolonged survival of the experimental animals after ^131^I and ^188^Re treatment 35.

To expand our strategies to include local as well as temporal control of *NIS* transgene induction and enhanced tumor selectivity of MSC-mediated *NIS* gene therapy, we engineered MSCs to express the *NIS* gene under control of a heat-inducible HSP70B promoter (HSP70B-NIS-MSCs).

Heat shock proteins (HSPs) are a heterogeneous group of molecular chaperones that includes the well-characterized 70-kDa HSP70 protein. The members of this family exhibit various cellular housekeeping and stress-related functions, such as the prevention of misaggregation, degradation, disaggregation and refolding of misfolded denatured proteins 45*.* Their synthesis can be induced within minutes in response to stress, such as heat, through the trimerization of heat shock factor-1 monomers that translocate to the nucleus where they bind to heat shock elements in target gene promoters, thereby activating a paused RNA polymerase II and allowing transcription to proceed (reviewed in 46)*.*

Among the different heat-responsive promoters tested for gene therapy, the human HSP70B promoter was found to have a relatively low background activity and allow a rapid high level of heat-induced transgene expression* in vitro* and *in vivo* (reviewed in 47). It was evaluated here as a candidate gene promoter for MSC-mediated *NIS* gene therapy. In the current study, we established and evaluated the use of a stable MSC line engineered with a heat-inducible HSP70B-NIS construct for enhanced control of tumor-specific *NIS* gene therapy.

## Materials and methods

### Plasmid constructs and stable transfection of MSCs

The plasmid construct pcDNA6.2ITRNEO- HSP70B-NIS, containing the full-length *NIS* gene (cDNA kindly provided by SM Jhiang, Ohio State University, Columbus, Ohio, USA) driven by the human HSP70B promoter, two sleeping beauty transposition sites and a geneticin resistance gene, was established as described previously 43 using the MultiSite Gateway Pro Plus Kit (Thermo Fisher Scientific, Waltham, Massachusetts, USA).

Simian virus 40 large T antigen-immortalized human bone marrow-derived MSCs were used for the experiments as the immortalized MSCs have been previously shown to retain the multilineage differentiation capacity, morphology and surface antigen pattern of primary MSCs but show greater expansion potential as aging and senescence are switched off 48. Transfection of MSCs was performed using the Neon Transfection System (Thermo Fisher Scientific) according to the manufacturer's instructions. Wild type MSCs (5 x 10^5^ cells) were electroporated with a total of 3 µg plasmid (pcDNA6.2ITRNEO-HSP70B-NIS plus pCMV(CAT) T7-SB100X, containing a sleeping beauty transposon system [provided by Z Ivics, Max Delbrück Center for Molecular Medicine, Berlin, Germany]) with a pulse voltage of 1300 Volt, a pulse width of 30 ms and a pulse number of 1. After 24 h incubation at 37 °C in a humidified CO_2_ incubator, selection medium was added containing 1% geneticin (G-418; Invitrogen, Carlsbad, California, USA). The clone showing the highest accumulation of radioiodide in an *in vitro* iodide uptake assay (see below), reflecting functional NIS expression, was used for further experiments (HSP70B-NIS-MSC).

### Cell Culture

Cells were cultured in an incubator at 37 °C, with 5% (v/v) CO_2_ atmosphere and 95% relative humidity. The human hepatocellular carcinoma (HCC) cell line HuH7 (JCRB0403; Japanese Collection of Research Bioresources Cell Bank, Osaka, Japan) was grown in Dulbecco's Modified Eagle Medium (1 g/l glucose; Sigma Aldrich, St. Louis, Missouri, USA) supplemented with 10% (v/v) fetal bovine serum (FBS; FBS Superior, Biochrom GmbH, Berlin, Germany) and 100 U/ml penicillin and 100 µg/ml streptomycin (P/S; Sigma-Aldrich). The human MSC line (HSP70B-NIS- MSC) was cultured in Roswell Park Memorial Institute (RPMI)-1640 culture medium (Sigma- Aldrich) enriched with 10% FBS, P/S and G-418.

### *In vitro* heat treatment

For the *in vitro* hyperthermia experiments, the cell culture dishes were sealed and the cells were exposed to heat at different temperatures ranging from 39 to 42 °C in a water bath for 30 to 60 min and then maintained in an incubator at 37 °C for 4 to 48 h.

### ^125^I uptake assay

NIS-mediated uptake of ^125^I in HSP70B-NIS- MSCs was measured as described previously 49, 50. Briefly, cells were seeded on 12-well plates and iodide uptake studies were performed at different time points (0 - 24 h) after heat treatment. Cells were incubated in Hanks' Balanced Salt solution (Gibco/Life Technologies, Carlsbad, California, USA), complemented with 10 µM NaI, 100 000 counts per minute (cpm) of Na^125^I/ml (PerkinElmer, Waltham, Massachusetts, USA) and 10 mM 4-(2-hydroxyethyl)- 1-piperazineethanesulfonic acid (HEPES; Sigma) (pH 7.3) for 45 min. The NIS-specific inhibitor KClO_4_ (100 mM; Merck Millipore, Burlington, Massachusetts, USA) was added to control wells to verify NIS specificity of uptake. After washing, cells were lysed in 1 N NaOH (Carl Roth GmbH + Co KG, Karlsruhe, Germany) for 15 min and trapped ^125^I was analyzed by γ-counting (Beckman Coulter GmbH, Krefeld, Germany). Results were normalized to cell survival (see below) and expressed as cpm / A620.

### Cell viability assay

The commercially available MTT (3-(4,5-dimethylthiazol-2-yl)-2,5-diphenyltetrazolium bromide) assay (Sigma Aldrich) was performed following the manufacturer's instructions. The absorbance of the resulting formazan product was measured on a Sunrise microplate absorbance reader (Tecan, Männedorf, Switzerland) at a wavelength of 620 nm using the software Magellan (Tecan).

### Quantitative real-time PCR

Total RNA of the heat treated and control HSP70B-NIS-MSCs was isolated 0 - 48 h after thermo-stimulation using the RNeasy Mini Kit with QIAshredder (Qiagen, Venlo, Netherlands) according to the manufacturer's recommendations. Single stranded cDNA was generated using Superscript III reverse transcriptase (Invitrogen). Quantitative real- time PCR (RT-PCR) was performed using the SYBR green PCR master mix (Qiagen) in a Mastercycler ep gradient S PCR cycler (Eppendorf, Hamburg, Germany). The following primers were used: *SLC5A5* (hNIS*)* (5´-TGCGGGACTTTGCAGTACATT-3´) and (5´-TGCAGATAATTCCGGTGGACA-3´), *HSPA1A* (5´-GATCAACGACGGAGACAAGC-3´) and (5´-GCTGCGAGTCGTTGAAGTAG-3´), *HSPA7* (5´-TTCCATGAAGTGGTTCACGA-3´) and (5´-TTGACGCTGGTGTCTTTGAG-3´), and *ACTB* (β-actin) (5´-AGAAAATCTGGCACCACACC-3´) and (5´-TAGCACAGCCTGGATAGCAA-3´). Levels of cDNA were normalized to the internal control β-actin. Relative expression levels were calculated using comparative ΔΔ-Ct values.

### Membrane preparation and Western blot

Membrane proteins from heat-treated and control cells were extracted as described previously 49 and protein concentration measured by Bradford assay (BioRad Laboratories Inc., Hercules, California, USA). Western blot analysis was conducted as reported previously 49, using a mouse monoclonal NIS-specific antibody (Merck Millipore; dilution 1: 1700) overnight at 4 °C and a horse-radish peroxidase- labeled goat anti-mouse antibody (Jackson ImmunoResearch Europe Ltd., Ely, UK; dilution 1:2000) for 1 h at room temperature. After 1 min incubation with enhanced chemiluminescence Western blotting detection reagent (WESTAR ETA C 2.0; Cyanagen Srl, Bologna, Italy), images were taken with an ECL ChemoCam Imager (INTAS, Göttingen, Germany). As control for protein loading, the membrane was stripped (Restore Western Blot Stripping Buffer, Thermo Fisher Scientific) and re-probed with a monoclonal anti-β-actin antibody produced in mouse (Sigma Aldrich; dilution 1:1500). The intensity of the bands was measured by densitometry using ImageJ software (NIH, Bethesda, Maryland, USA) and normalized to the β-actin loading control, expressed as the relative amount of NIS protein.

### Immunofluorescence staining

HSP70B-NIS-MSCs were seeded directly on FBS-coated microscope slides and grown until 60% confluent. 6 h after hyperthermia, the slides were air-dried overnight at room temperature and monolayers fixed with 80% methanol (Carl Roth) for 5 min at 4 °C, followed by 100% acetone (Carl Roth) for 2 min at -20 °C. Following blocking with 12% bovine serum albumin (Sigma-Aldrich) in phosphate- buffered saline (PBS; Sigma Aldrich) for 30 min, cells were then incubated with a primary mouse monoclonal NIS-specific antibody (Merck Millipore; dilution 1:500) for 90 min. A secondary Cy3 AffiniPure donkey anti-rabbit IgG antibody (Jackson ImmunoResearch; dilution 1:400) and bisbenzimide (Hoechst; Sigma Aldrich; dilution 1:2000) to counterstain nuclei were added for 30 min. Pictures were taken using an Axiovert 135 TV fluorescence microscope in combination with an AxioCam MRm CCD camera and the AxioVision Rel. 4.8 software (Carl Zeiss Microscopy GmbH, Jena, Germany).

### Establishment of subcutaneous HuH7 xenografts

5 - 6 week old female CD1 nu/nu mice were bought from Charles River (Sulzfeld, Germany) and kept under specific pathogen-free conditions with *ad libitum* access to water and mouse chow. The regional governmental commission for animals (Regierung von Oberbayern, Munich, Germany) authorized all experimental protocols.

Subcutaneous (s.c.) tumors were established by s.c. injection of 5 x 10^6^ HuH7 cells re-suspended in 100 µl PBS into the right flank region of the animals. Tumor volumes were determined using a caliper and calculated by the equation height x length x width x 0.52. When the tumor reached a volume > 1500 mm^3^ or showed signs of necrosis, mice were sacrificed.

### *In vivo* regional hyperthermia

For regional hyperthermia treatment* in vivo*, mice were anesthetized with inhalation isoflurane narcosis and placed on a water bath covered with a plastic plate that was specifically designed to allow only the tumor bearing leg to be submerged into the water through holes in the plastic cover. A rectal thermometer (Homeothermic Blanket Systems with Flexible Probe; Harvard Apparatus, Massachusetts, USA) monitored body temperature.

### Non-invasive monitoring of *in vivo* NIS biodistribution

As soon as tumors reached a volume of approximately 500 mm^3^, 5 x 10^5^ HSP70B-NIS-MSCs were injected systemically via the tail vein every second day for a total of three times. 3 days later, regional hyperthermia was applied (41 °C or, as control, 37 °C for 1 h). The mice received 18.5 MBq (0.5 mCi) of ^123^I (GE Healthcare Buchler GmBH & Co. KG, Braunschweig, Germany) intraperitoneally (i.p.) after 0, 6, 12, 18, 24, 36, 48, and 72 h and gamma camera imaging (e.cam, Siemens, Munich, Germany) was performed using a low-energy, high-resolution collimator. Intrinsic thyroidal iodide uptake was reduced by the addition of 5 mg/ml L-thyroxine (L-T4; Sigma Aldrich) to the drinking water ten days before ^123^I administration. Using the HERMES GOLD (Hermes Medical Solutions, Stockholm, Sweden) software, regions of interest were evaluated and tumoral iodide uptake was calculated and expressed as percentage of injected dose (ID) per tumor (% ID/tumor). Using the Medical Internal Radiation Dose (MIRD) concept, dosimetry was calculated with a RADAR dose factor (www.doseinfo-radar.com).

### *Ex vivo* immunohistochemical NIS protein staining

Paraffin-embedded tumor sections and a series of control organs from the mice used for ^123^I- scintigraphy were immunohistochemically stained as described previously 51. Staining was performed using a primary mouse monoclonal NIS-specific antibody (Merck Millipore; dilution 1:500) for 90 min followed by a biotin-SP-conjugated goat anti-mouse IgG antibody (Jackson Immunoresearch; dilution 1:200) for 20 min and then peroxidase-conjugated streptavidin (Jackson Immunoresearch; dilution 1:300) for an additional 20 min. Immunohistochemically stained tumor sections were scanned using the Pannoramic MIDI digital slide scanner and pictures taken with Caseviewer software (3DHISTECH Ltd., Budapest, Hungary) and control organs were imaged on an Olympus BX41 microscope equipped with an Olympus XC30 CCD camera (Olympus, Shimjukum Tokio, Japan).

### *Ex vivo* mRNA expression analysis

Frozen tumor sections of the ^123^I-scintigraphy were shredded using 20G and 25G syringes and RNA was isolated using the RNeasy Mini Kit with QIAshredder (see above). RT-PCR was run on a Lightcycler 96 System (Roche, Basel, Switzerland) and levels of cDNA were normalized to the mean of the internal controls β-actin, r18S and UBC. In addition to *NIS*, the primers r18S (5´-CAGCCACCCGAGATTGAGCA-3´) and (5´-TAGTAGCGACGGGCGGTGTG-3´) and UBC (5´-ATTTGGGTCGCGGTTCTTG-3´) and (5´-TGCCTTGACATTCTCGATGGT-3´) were used.

### ^131^I-therapy study

Therapy trials were started when tumors had an average size of 5 x 5 mm. Ten days before radioiodide injection, drinking water of mice was supplemented with L-T4 and standard mouse chow was switched to a low iodine diet (ssniff Spezialdiäten GmbH, Soest, Germany) to reduce radioiodine uptake by the thyroid gland and enhance potential tumoral iodine accumulation. Animals were randomly assigned to six treatment groups. HSP70B-NIS-MSCs were injected intravenously (i.v.) three times on every second day, followed by hyperthermia application 72 h later. 12 - 18 h after the heat treatment, 55.5 MBq (1.5 mCi) ^131^I (GE Healthcare) were injected i.p. This cycle was repeated 24 h after ^131^I administration, for a total of three times, with the second and third cycle consisting of only one MSC injection to reduce therapy duration (therapy group: HSP70B-NIS-MSCs + 41 °C + ^131^I; *n* = 9). To investigate the potential direct effects of hyperthermia on MSCs, we used saline instead of ^131^I (NaCl; Fresenius Kabi, Bad Homburg, Germany; HSP70B-NIS-MSCs + 41 °C + NaCl; *n* = 7). In an additional control group, MSCs and radioiodide were replaced by saline (NaCl + 41 °C + NaCl; *n* = 10) to investigate the potential effects of hyperthermia alone. As controls, the therapy schemes as described above were also conducted with 37 °C instead of 41 °C (HSP70B-NIS-MSCs + 37 °C + ^131^I; *n*=10, HSP70B-NIS- MSCs +3 7 °C + NaCl;* n*=8, and NaCl + 37 °C + NaCl; *n*=9). The tumor volume of the mice was estimated as described above and mice were sacrificed when the tumor volume exceeded 1500 mm^3^.

### *Ex vivo* immunofluorescence assay

Immunofluorescence staining on dissected frozen tissue samples of HuH7 tumors and quantitative analysis of cellular proliferation (Ki67; Abcam, Cambridge, UK; dilution 1:200) and blood vessel density (CD31; BD Pharmingen, Heidelberg, Germany; dilution 1:100) were performed according to the protocol described previously 28. Pictures were taken using a Leica DMI6000B microscope equipped with a Leica DFC365 FX camera and Leica MM AF software (Leica Microsystems GmbH, Wetzlar, Germany). Quantification of six visual fields per tumor section was performed using ImageJ software.

### Statistical analysis

All *in vitro* experiments were performed at least in triplicate and results are reported as mean ± standard error of the mean (SEM), mean fold change ± SEM or in percent. Statistical significance was tested by two-tailed Student's t-test, one-way analysis of variance (ANOVA) followed by post-hoc Tukey (honestly significant difference) test for comparison of more than two groups, two-way ANOVA followed by post-hoc Tukey test for the repeated measurements in the imaging study, or by log-rank for Kaplan-Meier survival plots. *p* values < 0.05 were considered significant (*p < 0.05; **p < 0.01; ***p < 0.001).

## Results

### *In vitro* radioiodide uptake studies of HSP70B-NIS-MSCs

**Figure 1:**
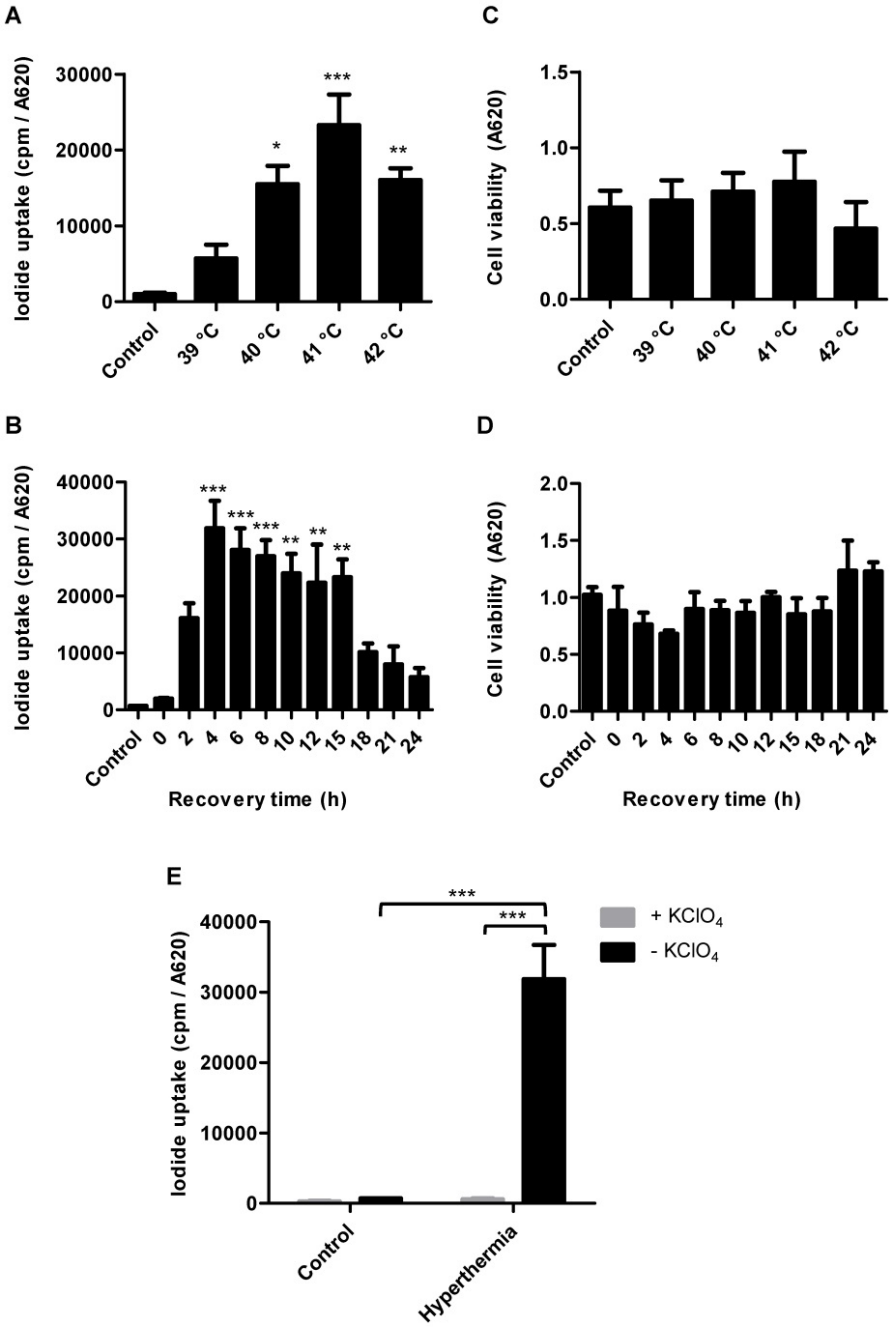
***In vitro* radioiodide uptake studies of HSP70B-NIS-MSCs.** HSP70B-NIS-MSCs were heat-treated in a water bath at temperatures ranging from 39 to 42 °C for 60 min, or as control at 37 °C, and functional NIS expression was analyzed by an *in vitro* iodide uptake assay (**A**). ^125^I uptake assay was performed at different time points (0 - 24 h) after promoter activation by heating the cells at 41 °C for 60 min (**B**). Cell viability after thermo-stimulation was analyzed by MTT assay (**C** +** D**). The NIS-specific inhibitor perchlorate was added to control the NIS dependency, analyzed 4 h after hyperthermia treatment and compared to unheated HSP70B-NIS-MSC control cells (**E**). Data are represented as mean ± SEM (*n* = 3; one way ANOVA analysis; *p < 0.05; **p < 0.01; ***p < 0.001).

Radioiodide uptake assays were performed to verify functional NIS expression by HSP70B-NIS- MSCs. The HSP70B promoter showed a low basal activity (no ^125^I uptake above background levels of unheated HSP70B-NIS-MSCs control cells) *in vitro* but showed a significantly induced expression in response to heat. Testing a temperature range from 39 to 42 °C, we observed the strongest radioiodine accumulation at 41 °C (Figure 1A). HSP70B-NIS-MSCs tolerated temperatures up to 41 °C well, but showed reduced viability yielding 71% viable cells at 42 °C (Figure 1C). In addition to temperature- dependence of NIS induction, we also characterized the time dependence of functional iodide uptake. The induction of ^125^I accumulation was found to occur rapidly after heat exposure with a maximum level reached as early as 4 h after hyperthermia application showing a 46-fold induction of iodide uptake as compared to unheated HSP70B-NIS-MSC-control cells (Figure 1B). The increase in iodide accumulation activity displayed a plateau with high uptake levels remaining for up to 15 h after heat-induced promoter activation. No significant change in cell viability was observed in response to heat in this time frame (Figure 1D). As a control, the iodide uptake was shown to be sensitive to the NIS-specific inhibitor perchlorate, demonstrating NIS dependency (Figure 1E).

### *In vitro* NIS mRNA and protein levels of HSP70B-NIS-MSCs

**Figure 2:**
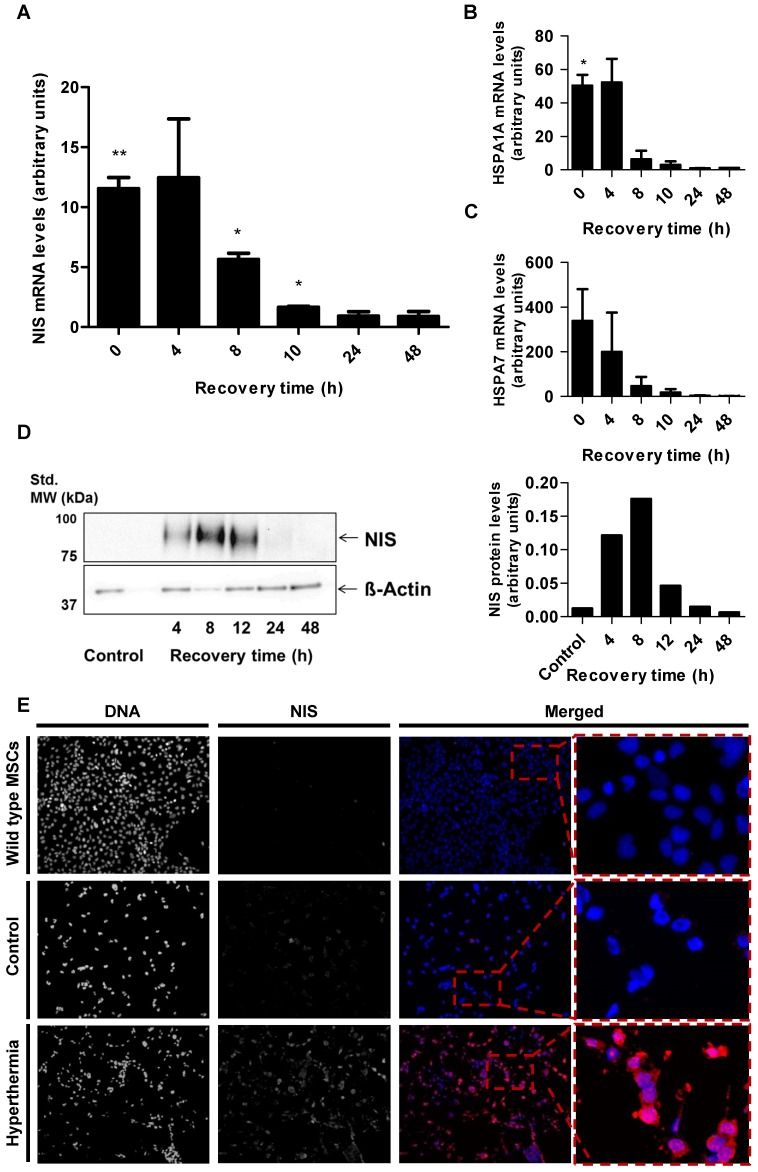
*** In vitro NIS* mRNA and protein levels of HSP70B-NIS-MSCs.** RT-PCR analysis of mRNA extracted from heat-treated (60 min at 41 °C) and unheated HSP70B-NIS-MSCs after 0 - 48 h, using primers for NIS (*SLC5A5*) (**A**), the endogenous HSP70 (*HSPA1A*) (**B**) and HSP70B (*HSPA7*) (**C**). Results were normalized to β-actin (*ACTB*), heat-treated compared to unheated MSCs for each time point and expressed as the mean ± SEM (*n* = 3; two-tailed Student's t-test; *p < 0.05; **p < 0.01). NIS protein levels were assessed by Western blot (**D**), extracted by membrane isolation at 4 - 48 h after thermo-stimulation or, as controls, of unheated HSP70B-NIS-MSCs. The intensity of the bands was measured by densitometry and normalized to the β-actin loading control. NIS-specific immunofluorescence staining (red) of heat-treated HSP70B-NIS-MSCs (bottom row), unheated HSP70B-NIS-MSCs (middle row), and unheated wild-type MSCs (top row) (**E**).

Parallel analysis of NIS mRNA levels by RT-PCR (Figure 2A) confirmed heat-induced and time- dependent *NIS* expression with the highest mRNA levels measured 4 h after heat treatment. Induction of mRNA expression of endogenous HSPs HSP70 (HSPA1A) (Figure 2B) and HSP70B (HSPA7) (Figure 2C) occurred in the same time frame as that seen for the *NIS* transgene. Western blot analysis showed a similar pattern of NIS protein expression with maximum protein levels seen 8 h after thermo- stimulation (Figure 2D). In addition, validation using immunofluorescence staining showed significant NIS-specific immunofluorescence in HSP70B-NIS- MSCs after hyperthermic treatment whereas only weak immunoreactivity was observed in non-heated, control HSP70B-NIS-MSCs, and no expression was seen in wild type MSCs (Figure 2E).

### Radioiodide biodistribution *in vivo* after MSC-mediated NIS gene transfer

By placing a thermo probe intratumorally, we confirmed rapid, stable and sufficient tumoral temperature (41.0 ± 0.1 °C or, as control, 37.0 ± 0.1 °C respectively) delivery, while the body temperature of the mice stayed within physiological levels [data not shown]. Following these studies, subsequent analyses were conducted without the invasive tumoral probe, which could distort results as the wound caused by the probe could potentially influence MSC recruitment.

**Figure 3:**
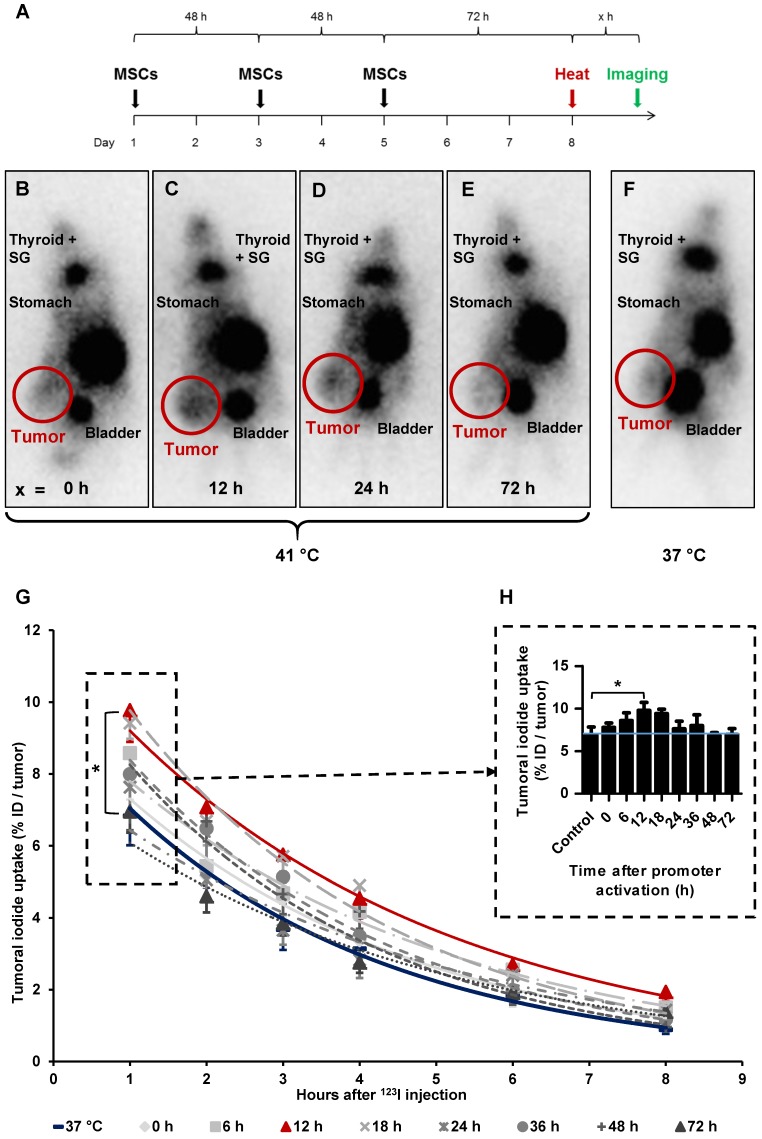
** Radioiodide biodistribution *in vivo* after MSC-mediated *NIS* gene transfer.** *In vivo,* using the hepatocellular carcinoma (HuH7) xenograft mouse model, HSP70B-NIS-MSCs were injected into the tail vein of mice, followed by hyperthermia, or as controls normothermia at 37 °C, 3 days later. 0 - 72 h after promoter activation by thermo-stimulation, 18.5 MBq ^123^I were injected and serial gamma camera imaging started (**A**). Images of gamma camera imaging taken 2 h after radioiodide of animals with 0 h (**B**; *n* = 7), 6 h (*n* = 5), 12 h (**C**; *n* = 7), 18 h (*n* = 4), 24 h (**D**; *n* = 6), 36 h (*n* = 4), 48 h (*n* = 5) and 72 h (**E**; *n* = 4) between promoter activation by hyperthermia and radioiodine injection and control animals, treated at 37 °C (**F**; *n* = 5) (one representative image for each treatment group). Quantification of serial ^123^I-scintigraphy (**G**) representing the efflux of the injected ^123^I and comparison of the tumoral ^123^I accumulation 1 h post injection (**H**) (the blue line represents the controls at 37 °C). Results are expressed as mean ± SEM; two-way ANOVA with post-hoc Tukey test *p < 0.05.

After three HSP70B-NIS-MSCs injections, tumor- bearing mice were heat-treated regionally for 1 h at 41 °C, to activate the heat-inducible HSP70B promoter, or as control at 37 °C. 0 to 72 h later, 18.5 MBq ^123^I were administrated and functional NIS expression was analyzed by gamma camera imaging. The images of the ^123^I-scintigraphy revealed the strongest tumoral iodine accumulation, mediated by functional NIS, in animals with a latency of 12 h (Figure 3C) between hyperthermia and radioiodine injection, whereas mice in the 37 °C control group (Figure 3F) exhibited the weakest signal. Quantitative analysis of serial scanning (Figure 3G) showed no difference to 37 °C control levels (maximum of tumoral iodide uptake 1 h post ^123^I injection [Figure 3H]: 37 °C control 6.83 ± 1.99% ID/tumor) when radioiodine was injected directly after thermo-stimulation (Figure 3B, G-H; 0 h 7.78 ± 1.30% ID/tumor). However, a slight increase of tumoral iodine accumulation was observed (Figure 3 G-H; 8.58 ± 1.86% ID/tumor) when there was a period of 6 h between hyperthermia and ^123^I injection. Significantly increased and maximal radioiodine levels were reached at the 12 h interval between promoter activation by heat treatment and radioiodide administration (Figure 3C,G-H; 9.77 ± 2.33% ID/tumor) with a plateau up to 18 h (Figure 3G-H; 9.41 ± 0.86% ID/tumor). All other groups, with a gap of 24 h (Figure 3D,G-H;7.78 ± 0.85% ID/tumor), 36 h (Figure 3G-H; 7.99 ± 2.22% ID/tumor), 48 h (Figure 3G-H; 6.79 ± 0.79% ID/tumor) and 72 h (Figure 3E,G-H; 6.96 ± 1.18% ID/tumor) between HSP70B stimulation and imaging exhibited a similar iodine uptake as the control group. Physiological radioiodide accumulation in the thyroid and salivary glands (SG), stomach and, due to renal elimination of ^123^I, in the urinary bladder, was visible in all animals (Figure 3B-F). Dosimetric calculations revealed a higher tumor-absorbed dose of 70 ± 28 mGy/MBq/g tumor and an effective half-life of 3.6 h for ^131^I in heat-treated animals with the 12 h interval, as compared to the unheated control group with 44 ± 15 mGy/MBq/g tumor-absorbed dose and a calculated effective half-life of 3.2 h for ^131^I.

### *Ex vivo* analysis of NIS expression

**Figure 4:**
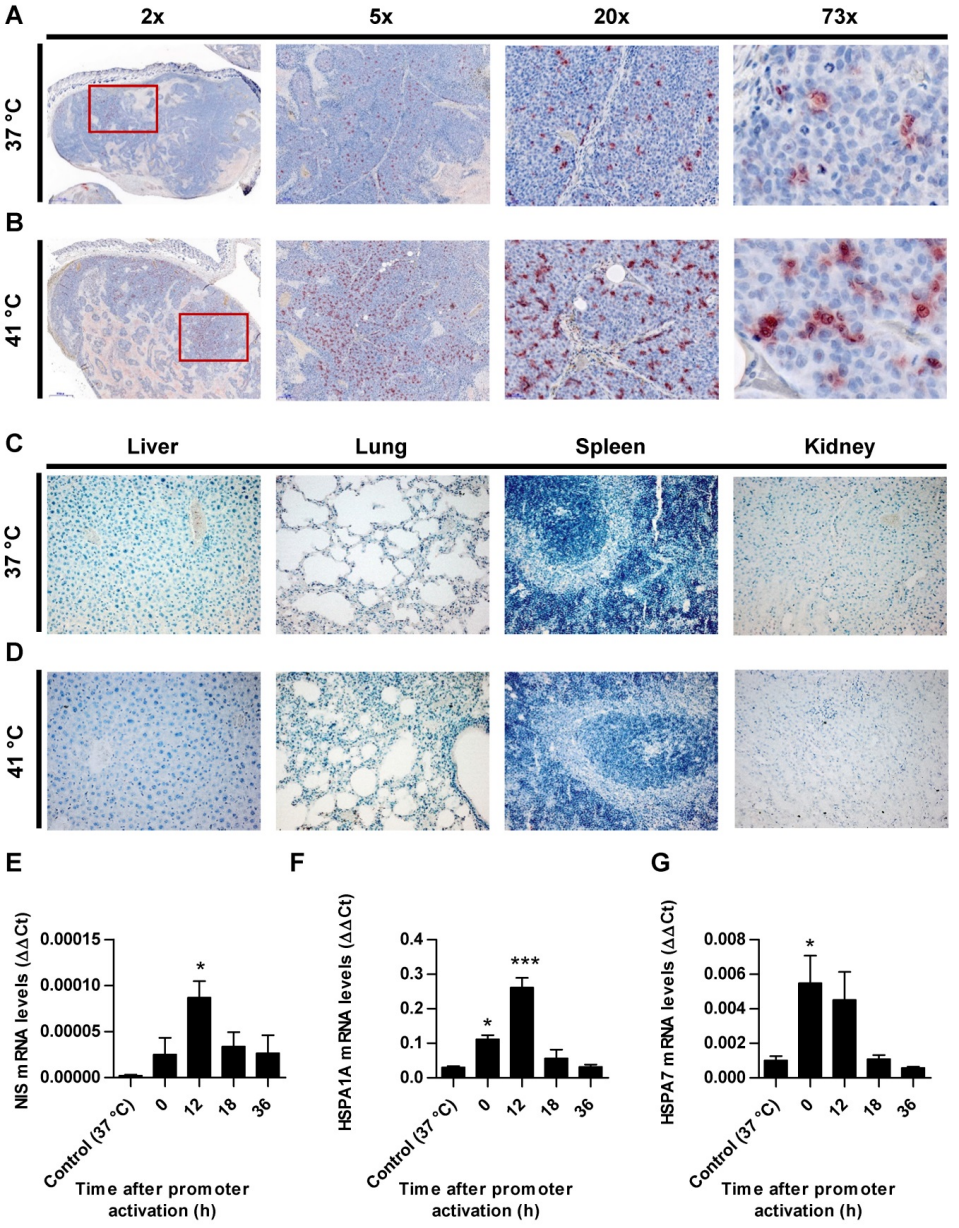
*** Ex vivo* analysis of NIS expression.** NIS-specific immunohistochemistry (red) was performed on paraffin-embedded HuH7 tumor sections. Tumors of mice of 37 °C controls (**A**) compared to mice which were heat-treated with a 12 h latency between promoter activation and ^123^I administration (**B**). Control organs (liver, lung, spleen, and kidney) of mice receiving 37 °C (**C**) or 41 °C (**D**) treatment. One representative image is shown each at 2x - 73x magnification for tumor sections and 20x for control organs. mRNA was isolated from frozen tumors sections of heat treated mice (groups in which 0, 12, 18, and 36 h were in between promoter activation and start of ^123^I-scintigraphy) and controls (37 °C) at the end of the serial gamma camera imaging and analyzed for *NIS* (**E**), endogenous *HSPA1A* (**F**) and *HSPA7* (**G**) by RT-PCR (*n* = 4; one-way ANOVA; *p < 0.05, ***p < 0.001).

Paraffin-embedded tissue sections derived from HuH7 xenografts after ^123^I-scintigraphy were stained immunohistochemically using a monoclonal NIS antibody. Tumor sections from control animals (37 °C; Figure 4A) showed much less perivascular human NIS-specific immunoreaction (red) as did the group of thermo-stimulated animals (41 °C, 12 h gap; Figure 4B). Control organs (liver, lungs, spleen and kidney) of heat-treated (Figure 4D) and control animals (Figure 4C) did not show NIS-specific immunoreactivity. Frozen tumor sections were processed for mRNA isolation. RT-PCR showed an upregulation of NIS (Figure 4E), as well as the endogenous HSPs, HSP70 (Figure 4F) and HSP70B (Figure 4G) in a temperature- and time-dependent fashion as an additional confirmation of the functional NIS expression as well as the efficient heat transfer to the tumors.

### MSC-mediated NIS gene therapy *in vivo*

**Figure 5:**
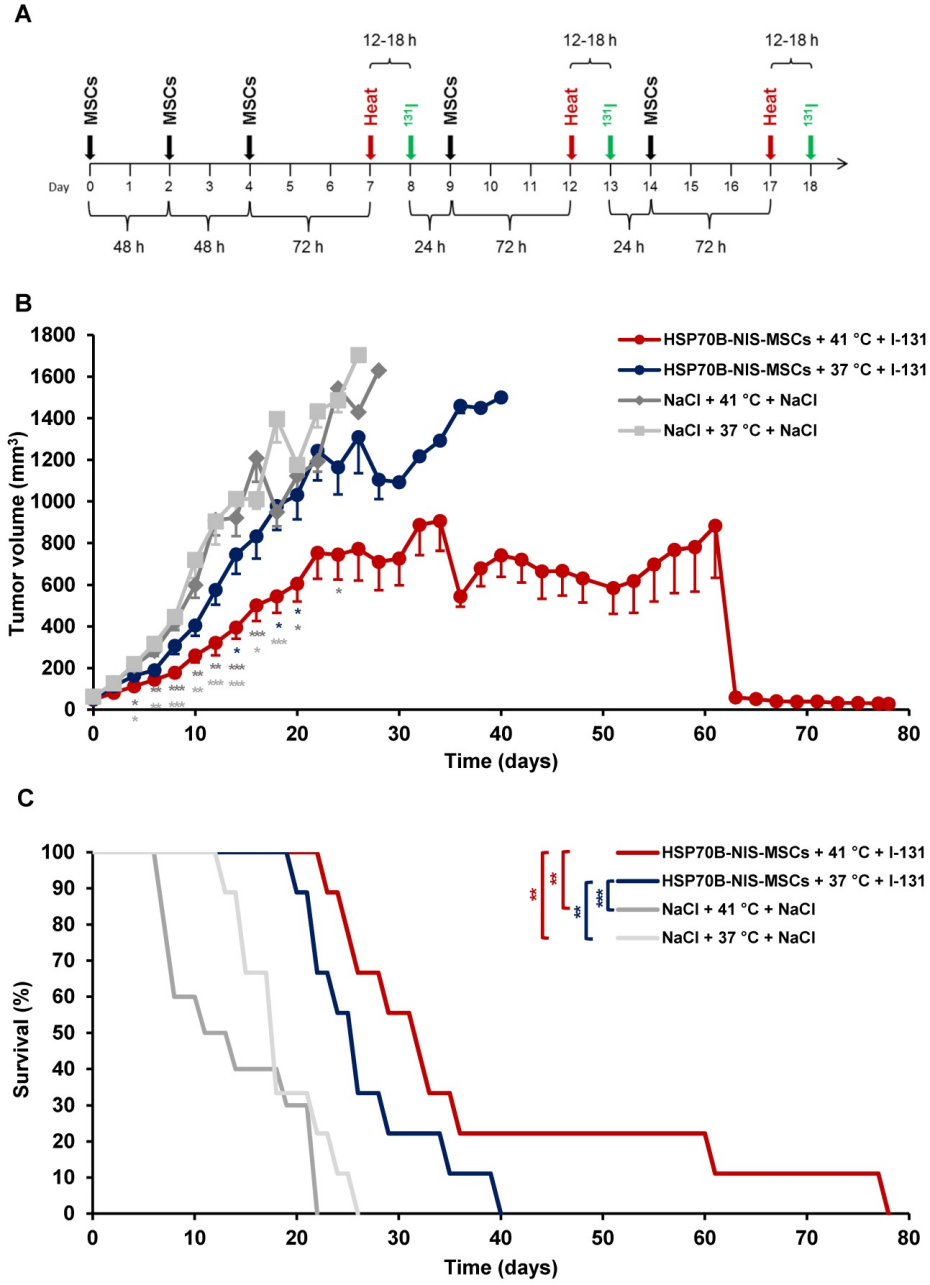
** Heat-induced MSC-mediated *NIS* gene therapy *in vivo.***Three days after systemic injections of HSP70B-NIS-MSCs (black arrows), hyperthermia (red arrows) was administered to mice harboring HuH7 xenograft tumors. 12 to 18 h later, 55.5 MBq of therapeutic ^131^I (green arrows) was applied. This treatment cycle was repeated for a total of three times (**A**). Tumor growth (**B**) and overall survival (**C**) were evaluated for the treatment with HSP70B-NIS-MSCs, hyperthermia and ^131^I (HSP-NIS-MSCs + 41 °C + ^131^I; *n* = 9), compared to control groups, receiving hyperthermia and saline (NaCl + 41 °C + NaCl; *n* = 10) and to normothermic groups (HSP70B-NIS-MSCs + 37 °C + ^131^I; *n* = 9 and NaCl + 37 °C + NaCl; *n* = 10). Results are expressed as mean ± SEM (one-way ANOVA for tumor growth and log-rank test for Kaplan-Meier survival plots; *p < 0.05; **p < 0.01; ***p < 0.001).

Following validation of functional NIS expression by non-invasive radioiodine imaging, a therapy trial with ^131^I was initiated based on the approach that showed optimal tumoral iodine accumulation as seen by the ^123^I-scintigraphy. After three MSC administrations, radioiodine was injected 12 to 18 h after hyperthermic treatment to be within the plateau phase as seen *in vitro* and by gamma camera imaging. This cycle was repeated for a total of three times, but cycle two and three consisted of only one MSC application to reduce the overall length of the treatment scheme (Figure 5A). Mice injected with saline only with (41 °C) (NaCl + 41 °C + NaCl) or without hyperthermia (37 °C) (NaCl + 37 °C + NaCl), and mice receiving saline instead of ^131^I with (41 °C) (HSP70B-NIS-MSCs + 41 °C + NaCl) and without hyperthermia (37 °C) (HSP70B-NIS-MSCs + 37 °C + NaCl), served as controls and exhibited an uninterrupted and exponential tumor growth (Figure 5B and Supplementary Figure 1). Mice treated with HSP70B-NIS-MSCs and ^131^I following hyperthermia at 41 °C (HSP70B-NIS-MSCs + 41 °C + ^131^I) exhibited a significantly reduced tumor growth as compared to all control groups, which was associated with prolonged survival of the mice (Figure 5C). In one mouse, an impressive partial remission was observed with tumor shrinkage from 410 mm^3^ to 28 mm^3^ until day 78. As compared to the saline control groups, survival analysis also revealed a significantly prolonged survival of mice treated with HSP70B-NIS-MSCs of up to 78 days after therapy start in the hyperthermia group (HSP70B-NIS-MSCs + 41 °C + ^131^I) and up to 40 days (HSP70B-NIS-MSCs + 37 °C + ^131^I) in the normothermic group. At day 22 after the start of therapy, all animals within the NaCl + 41 °C + NaCl group had to be sacrificed based on tumor growth, whereas 100% of the HSP70B-NIS-MSCS + 41 °C + ^131^I and 70% of the HSP70B-NIS-MSCS + 37 °C + ^131^I groups were still alive. By day 26, all animals in the saline control groups had reached the endpoint criteria. The median survival after therapy start for the therapy group HSP70B-NIS-MSCs + 41 °C + ^131^I was 31 days, for the HSP70B-NIS-MSCs + 37 °C + ^131^I 25 days, for NaCl + 41 °C + NaCl treated animals 13 days, and for the NaCl + 37 °C + NaCl group 17 days. The treated mice showed no major adverse effects of radionuclide treatment and were sacrificed due to tumor load, only one mouse due to respiratory problems on day 78 after therapy start.

### *Ex vivo* proliferation and blood vessel density analysis

**Figure 6:**
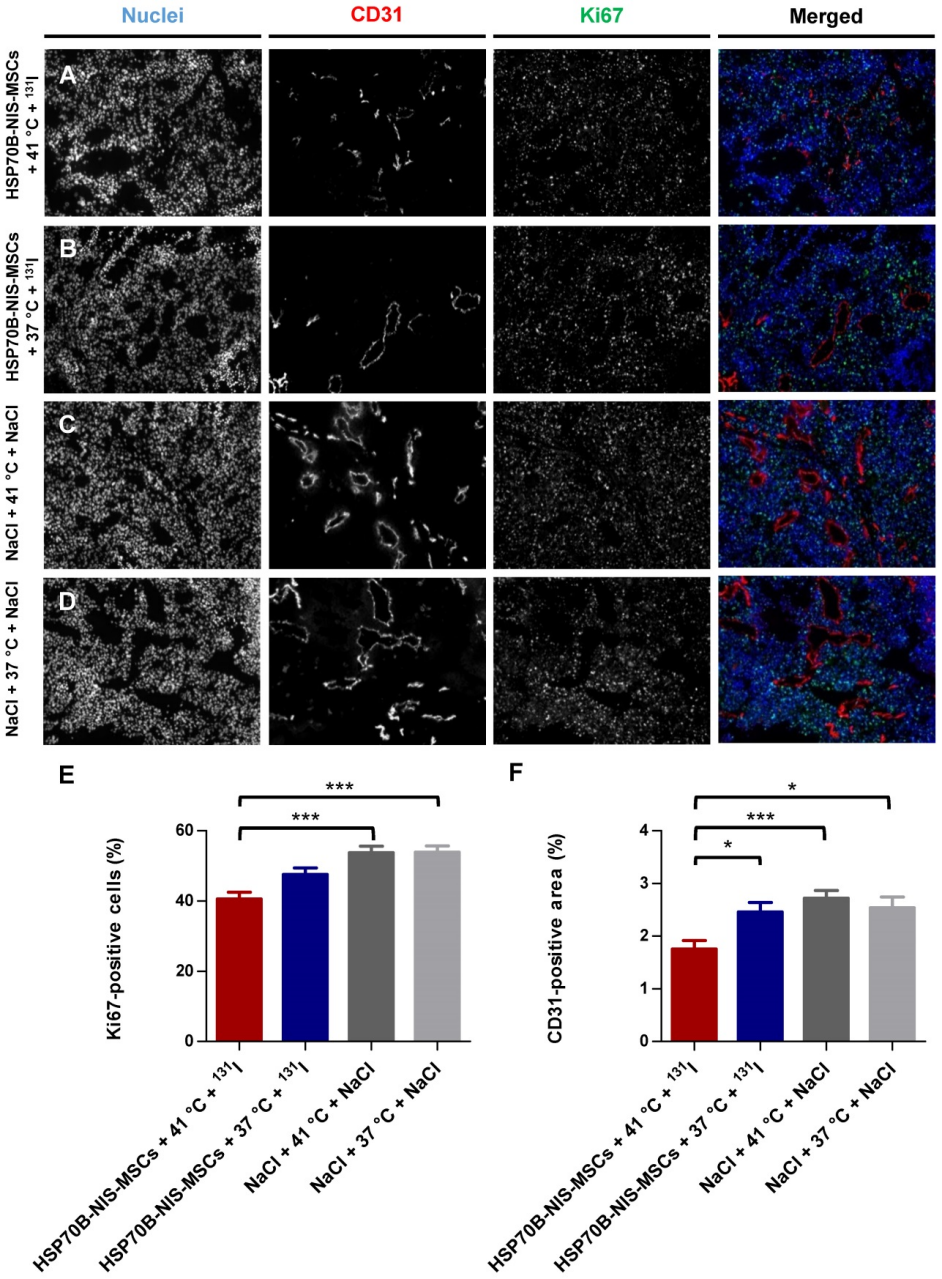
** Reduced *ex vivo* proliferation and blood vessel density as result of heat-induced MSC-mediated *NIS* gene therapy.** Ki67 [(**E**); green; proliferation index] and CD31 [(**F**); red; labeling blood vessels] immunofluorescence staining of frozen tumor sections was performed on tumors derived from mice receiving HSP70B-NIS-MSCs at the end of the ^131^I therapy (**A**; *n* = 9), unheated controls (**B**; *n* = 9) and mice receiving saline with (**C**; *n* = 10) or without (**D**; *n* = 9) heat treatment instead of MSCs. Nuclei were counterstained with Hoechst (blue). One representative image is shown each at 10x magnification. Results are expressed as mean ± SEM (one-way ANOVA; *p < 0.05; ***p < 0.001).

At the end of the observation period, the tumors were dissected and frozen tumor sections were stained using an antibody to identify blood vessels (CD31; red) and a Ki67-specific antibody to display general cell proliferation (green) (Figure 6A-D). Tumors treated with HSP70B-NIS-MSCs, radioiodide, and hyperthermia (HSP70B-NIS-MSCs + 41 °C + ^131^I) (Figure 6A) showed a significantly lower proliferation index as did the saline control groups (Figure 6E), as well as a significantly lower blood vessel density compared to all three control groups (Figure 6F), thereby demonstrating an antiangiogenic effect and thus further validating successful HSP70B-NIS-MSC- mediated ^131^I therapy.

## Discussion

When used as a theranostic gene, the symporter *NIS* acts as an effective molecular reporter gene with robust therapeutic options, thus enabling the visualization and treatment of tumors through the application of appropriate radionuclides. NIS efficiently transports various radionuclides, allowing the application of easily available and extensively studied radionuclides such as ^123^I, ^124^I, ^18^F-tetrafluoroborate or ^99m^Tc for *in vivo* imaging of functional NIS expression, and ^131^I, ^188^Re, or ^211^At for the delivery of therapeutic applications (summarized in 52-54). The treatment of thyroid cancer with ^131^I has been performed in the clinic with great success since 1946 with a well-understood safety profile and is still the most preferred treatment modality, after thyroidectomy, for differentiated thyroid cancer 2. The potential use of exogenously applied *NIS* to induce radioiodine accumulation in non-thyroidal tumors has been investigated in a variety of *in vitro* and *in vivo* tumor models, such as anaplastic thyroid cancer 55, glioma 22, acute myeloid leukemia 21, multiple myeloma 7, cancer of the prostate 6, 15, 16, 23, 56, the liver 37, the colon 27, 36, the breast 8, and the pancreas 19, 39, 57 by several groups including our own. These very promising proof-of- principle studies have led to phase I/II clinical studies with *NIS* as a reporter gene and / or therapy gene using virus-mediated *NIS* gene delivery for locally recurrent prostate cancer (NCT00788307), multiple myeloma (NCT00450814, NCT02192775, NCT03017820), and for various other non-thyroidal cancer types (NCT01503177, NCT01846091, NCT02364713, NCT02700230, NCT02919449, NCT02962167, NCT03120624, NCT03171493, NCT03647163) 58-61.

The ultimate goal of tumor gene therapy is the efficient delivery of a transgene by the use of systemically applied vectors. To this end, engineered versions of MSCs represent attractive candidates as gene delivery vehicles 32. MSCs are actively recruited to sites of tissue injuries or chronic inflammation and contribute to tissue remodeling 62. The body sees “tumors as wounds that do not heal” 63, 64 and for this reason it drives the mobilization of MSCs from various tissue stores and their subsequent migration to developing tumor stroma 65, 66. MSCs exhibited selective incorporation into growing tumor stroma 67, 68 driven by high local concentrations of inflammatory chemokines and cytokines secreted by the tumor and the tumor stroma 69, 70. This tumor tropism of MSCs has been used as a “Trojan Horse”-like therapy approach in which genetically engineered MSCs deliver a therapeutic agent, in our case the *NIS* gene, deeply into growing tumors 34-37, 39-41, 43, 71-74. The adaption of engineered MSCs as a therapeutic approach to treat solid cancers using NIS has proceeded towards clinical application with the initiation of a first series of phase I/II trials using measles virus (MV)-NIS transfected MSCs in recurrent ovarian cancer (NCT02068794). In addition, a clinical trial using MSCs as gene delivery vehicles based on our previous work was completed using MSCs modified with CCL5-driven HSV-TK for the treatment of gastrointestinal cancer 42. In additional clinical trials, researchers are evaluating IFN-β expressing MSCs in ovarian cancer (NCT02530047) at MD Anderson Cancer Center, and the safety and anti-tumor capacity of TRAIL-modified MSCs in metastatic non-small cell lung cancer (NCT03298763) at University College London Hospital.

While MSCs show significant tumor tropism, a portion of the adoptively applied MSCs potentially also home to normal tissues, such as the spleen or the lung 35. Therefore, selective control of the expression of the transgene provides a means of limiting potential damage to non-tumor tissues. Our group has demonstrated that this can be achieved by using specific gene promotors linked to signals or differentiation pathways that occur mostly or only within the tumor setting. We have previously shown that MSCs expressing *NIS* under control of the tumor stroma-specific RANTES/CCL5 promoter 35, a HIF-1α-driven synthetic promoter activated by tumor hypoxia 37, or using a synthetic promoter responsive to transforming growth factor B1 (TGFB1) present in the tumor setting 43 can lead to a robust and efficient NIS expression within tumors. In these settings, the biodistribution of our genetically engineered MSCs was analyzed by ^123^I-scintigrahy or ^124^I- and ^18^F-TFB PET-imaging, using *NIS* as reporter gene. The subsequent systemic application of ^131^I was shown for each approach to result in a significant reduction in tumor growth and a prolonged survival of the animals, thus demonstrating both the great potential of *NIS* as a theranostic gene and the potential benefit of engineered MSCs as therapy vehicles. External beam radiation was recently found to efficiently enhance MSC recruitment to treated tumors as well as to strongly increase tumor levels of TGFB1 40. Using a TGFB1-inducible SMAD- responsive promoter for MSC-mediated *NIS* gene therapy in combination with external beam radiation dramatically increased the therapeutic efficacy of *NIS* gene therapy with complete tumor remission seen in a subset of mice 41.

The present study builds on these results to evaluate a potentially more precise means of controlling theranostic transgene expression through the use of the heat-inducible HSP70B promoter that was shown here to confer local and temporal control of strong and robust induction of transgene expression. The HSP70B (*HSPA7*) gene was discovered in 1985 75, is encoded near the highly homologous HSP70B' (*HSPA6*) on chromosome 1 76, and, although mRNA is expressed after thermo- stimulation, it does not appear to encode a functional protein 77.

The heat-inducible HSP70B promoter was selected for use in our MSC-mediated *NIS* gene transfer approach. *In vitro* characterization of HSP70B- NIS-MSCs demonstrated a time- and temperature- dependent NIS-mediated iodine accumulation. The HSP70B promoter showed only background expression before heat was applied, but was significantly induced allowing a more than 45-fold functional NIS response to 60 min of heat treatment at 41 °C. NIS expression reached a maximum by 6 to 8 h after thermo-stimulation that was confirmed at mRNA and protein levels by RT-PCR, Western blot and NIS-specific immunofluorescence, respectively. These results are in line with previous studies that used the HSP70B promoter, where a similar pattern of temperature- and time-dependent promoter activity was observed* in vitro* 78-81.

We could validate these findings in an *in vivo* HCC xenograft mouse model using ^123^I-scintigraphy imaging studies that revealed a pattern of time-dependent tumoral iodine uptake, mediated by NIS, *in vivo* that closely matched our* in vitro* results. The strongest iodine uptake *in vivo* was found 12 h after hyperthermia treatment that was only slightly later than that seen in the cell culture studies (6 - 8 h after promoter activation). As seen with other HSP proteins that are often increased in tumor settings without thermo-stimulation due to the proteotoxic stressful conditions that cancer cells face, including nutrient deprivation, the presence of free reactive oxygen species, hypoxia, acidosis, and high levels of mutant proteins 82, 83, the HSP70B promoter (HSP70B-NIS-MSC) showed increased basal activity within the tumor microenvironment *in vivo* as evidenced by gamma camera images in the 37 °C control group. This parallels the observation that HSP70 is typically found to be increased in tumors 84 and is currently evaluated as tumor-specific diagnostic and therapeutic target 85. Importantly, the HSP70B-NIS-MSCs were not found to be activated in non-target organs (Figure 4 C + D).

Therapy using hyperthermia and ^131^I led to a strongly reduced tumor growth, prolonged survival with reduced blood vessel density and proliferation index, demonstrating the long-term antiangiogenic therapeutic efficacy of ^131^I. At normothermic conditions (37 °C), treatment with HSP70B-NIS-MSCs followed by ^131^I application resulted in slightly reduced tumor growth (22% showed a response to the therapy). HSP70B-NIS-MSCs, ^131^I and hyperthermia (41 °C) treatment resulted in a robust and significant therapeutic effect with reduced tumor growth in 67% of the animals, and a partial tumor remission in one animal. Hyperthermia and MSCs alone (HSP70B-NIS- MSCs + 41°C + NaCl) showed no difference in survival or tumor growth (Supplementary Figure 1 A +B) compared to unheated controls (HSP70B-NIS- MSCs + 37 °C + NaCl). Damage to the thyroid as well as the salivary glands is a potential and well- documented side effect of radioiodide therapy. In the present study, we used protocols similar to those used for patients that downregulate NIS expression in the thyroid gland (which is TSH regulated) by L-T4 pretreatment that helps to limit the damage to the thyroid and increases the circulating levels of radioiodide. Most of the iodide uptake in the cervical region seen on the images in Figure 3 is caused by iodide uptake in the salivary glands (that is not TSH regulated) which is anatomically close to the thyroid gland in mice and much bigger in comparison to the thyroid gland in humans 86. Xerostomia is a recognized side effect of radioiodine treatment in some patients, but importantly, it is clinically manageable and has to be weighed against the benefit of effective tumor growth control.

One of the limitations of the present study is the somewhat heterogeneous tumoral response to hyperthermia. The therapy worked well in most of the animals, but in 33% of the animals the effect was reduced. This is believed to be due in part to the general model system applied here that used a water-based regional heat transfer method that has limitations for homogenous heat application in mouse flank tumors. This should be less of an issue in the clinic as the next generation hybrid magnetic resonance-guided high-intensity focused ultrasound now being used for tumor hyperthermia therapy allows a highly focused heating of the region with real-time temperature mapping and energy deposition 87.

As heat shows great chemo- and radiosensitizing qualities, hyperthermia has high potential as an adjuvant in multimodal treatment approaches, especially for sarcoma, melanoma, breast, or colon cancer 87. The therapeutic effects of chemo- or radiotherapy can be enhanced by the administration of hyperthermia. Mild hyperthermia, at temperatures in the range of 39 to 44 °C, is able to disturb the *de novo* synthesis of DNA by denaturation of synthetases and polymerases resulting in arrest of the cell cycle. It can furthermore induce apoptosis or necrosis, and interfere in a number of DNA repair mechanisms 88, 89. Recently, there is also great interest in hyperthermia in the context of oncological immunotherapy, especially in regards to the therapy of metastases. In particularly, as hyperthermia can activate immune cells, initiate the release of exosomes and HSPs that present tumor antigens, enhance surface molecule expression on heated tumor cells, and thereby increase the immune sensitivity of tumors cells to the immune system. Lately, a phenomenon, called the abscopal effect, that was first discovered for local radiotherapy was now also detected for hyperthermia. It describes the findings that local tumor treatment is able to affect the growth of distant tumors and metastases and it is presumably mediated by the activation of the immune system 90.

In summary, our data demonstrate the potential of using a heat-inducible promoter (HSP70B) to drive NIS expression in MSCs, which allow an increased tumor-specific, temperature- and time-dependent NIS-mediated accumulation of radioiodide in heat-treated tumors. Application of ^131^I led to significantly reduced tumor growth and prolonged survival of animals receiving HSP70B-NIS-MSCs, ^131^I and hyperthermia. This proof-of-principle study has opened a new and exciting chapter on the way towards a future clinical application of genetically engineered MSCs in the context of *NIS* gene therapy with high translational relevance.

## Supplementary Material

Supplementary figure.

## Abbreviations

ANOVA: analysis of variances
CCL5: CC- chemokine ligand 5
cDNA: complementary DNA
CMV: cytomegalovirus
cpm: counts per min
DNA: deoxyribonucleic acid
FBS: fetal bovine serum
HCC: hepatocellular carcinoma
HIF1α: hypoxia-inducible factor 1α
HSP: heat shock protein
HSV-TK: herpes simplex virus-thymidine kinase
ID: injected dose
i.p.: intraperitoneal
i.v.: intravenous
L-T4: L-thyroxine
MSCs: mesenchymal stem cells
mRNA: messenger ribonucleic acid
MTT: 3-(4,5-dimethylthiazol-2-yl)- 2,5-diphenyltetrazolium bromide
NaCl: saline
NIS: sodium iodide symporter
PBS: phosphate-buffered saline
PET: positron-emission tomography
RANTES: regulated on activation, normal T cell expressed and secreted
RNA: ribonucleic acid
RT-PCR: real-time polymerase chain reaction
s.c.: subcutaneous
SEM: standard error of the mean
SG: salivary glands
TGFB1: transforming growth factor beta 1
