# Supplementary material for: Effective control of tumor growth through spatial and temporal control of theranostic sodium iodide symporter (NIS) gene expression using a heat-inducible gene promoter in engineered mesenchymal stem cells
Source: Theranostics. 2020 Mar 15;10(10):4490–506. doi: 10.7150/thno.41489 (PMC7150485; doi:10.7150/thno.41489)
Supplement: Supplementary file 1 — Supplementary figure. [file thnov10p4490s1.pdf]

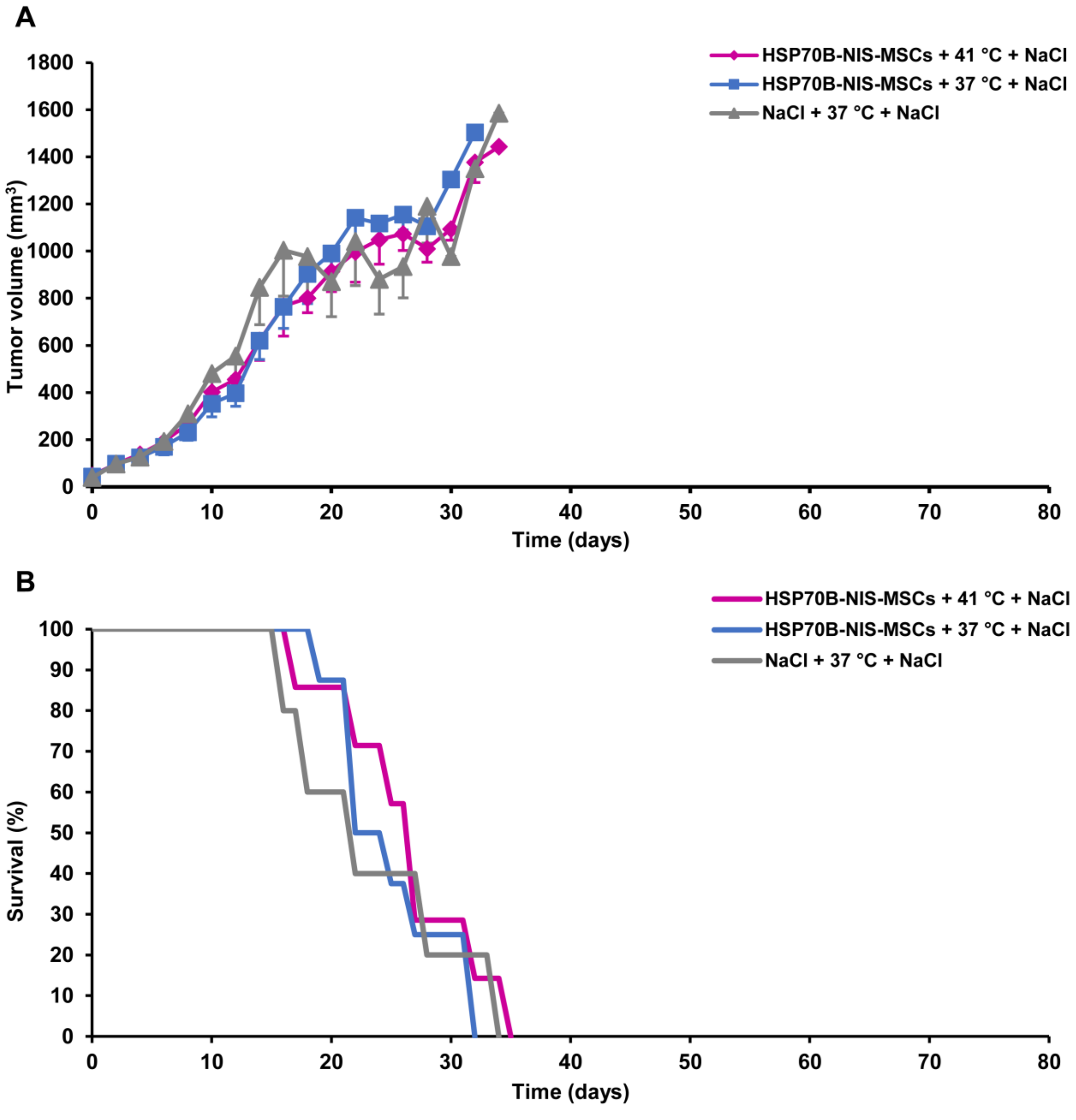

Supplementary Figure 1: Control groups for heat-induced MSC-mediated *NIS* gene therapy *in vivo*

Three days after systemic injections of HSP70B-NIS-MSCs, hyperthermia was administered to mice harboring HuH7 xenograft tumors. 12 to 18 h later, saline was applied instead of ^{131}I as control. This treatment cycle was repeated for a total of three times. Tumor growth (A) and overall survival (B) were evaluated for the treatment with HSP70B-NIS-MSCs, hyperthermia, and NaCl (HSP-NIS-MSCs + 41 °C + NaCl; *n* = 7), compared to a normothermic control group (HSP70B-NIS-MSCs + 37 °C +

NaCl; *n* = 8), and saline control group (NaCl + 37 °C + NaCl; *n* = 5). Results are expressed as mean ± SEM.
